# Biotransformation of Protopanaxadiol-Type Ginsenosides in Korean Ginseng Extract into Food-Available Compound K by an Extracellular Enzyme from *Aspergillus niger*

**DOI:** 10.4014/jmb.2007.07003

**Published:** 2020-07-31

**Authors:** Eun-Bi Jeong, Se-A Kim, Kyung-Chul Shin, Deok-Kun Oh

**Affiliations:** Department of Bioscience and Biotechnology, Konkuk University, Seoul 05029, Republic of Korea

**Keywords:** *Aspergillus niger*, biotransformation, compound K, protopanaxadiol ginsenosides, *Panax ginseng*

## Abstract

Compound K (C-K) is one of the most pharmaceutically effective ginsenosides, but it is absent in natural ginseng. However, C-K can be obtained through the hydrolysis of protopanaxadiol-type ginsenosides (PPDGs) in natural ginseng. The aim of this study was to obtain the high concentration of food-available C-K using PPDGs in Korean ginseng extract by an extracellular enzyme from *Aspergillus niger* KACC 46495. *A. niger* was cultivated in the culture medium containing the inducer carboxymethyl cellulose (CMC) for 6 days. The extracellular enzyme extracted from *A. niger* was prepared from the culture broth by filtration, ammonium sulfate, and dialysis. The extracellular enzyme was used for C-K production using PPDGs. The glycoside-hydrolyzing pathways for converting PPDGs into C-K by the extracellular enzyme were Rb1 → Rd → F2 → C-K, Rb2 → Rd or compound O → F2 or compound Y → C-K, and Rc → Rd or compound Mc1 → F2 or compound Mc → C-K. The extracellular enzyme from *A. niger* at 8.0 mg/ml, which was obtained by the induction of CMC during the cultivation, converted 6.0 mg/ml (5.6 mM) PPDGs in Korean ginseng extract into 2.8 mg/ml (4.5 mM) food-available C-K in 9 h, with a productivity of 313 mg/l/h and a molar conversion of 80%. To the best of our knowledge, the productivity and concentration of C-K of the extracellular enzyme are the highest among those by crude enzymes from wild-type microorganisms.

## Introduction

The root of Korean ginseng (*Panax ginseng* Meyer) have been used as traditional herbs to treat diseases and maintain for thousands of years in Asian countries such as Korea, China, and Japan. Ginsenosides are the main components in ginseng for pharmacological effects. They are divided into protopanaxatriol-type ginsenosides (PPTGs) and protopanaxadiol-type ginsenosides (PPDGs) according to the position and number of their hydroxyl groups. Major ginsenoside such as Rb1, Rb2, Rc, Rd, Re, and Rg1 comprise more than 80% of the ginsenosides in wild ginseng. Minor ginsenosides such as F2, Rg3, Rh1, Rh2, and compound K are the deglycosylated from of the major ginsenosides [1]. These are known to be more bioactive than the major ginsenosides because of their improved permeability across the cell membrane in the gastrointestinal tract. Thus, major ginsenosides are needed to be transformed into minor ginsenosides by the hydrolysis of the saccharide moiety in the ginsenosides [2].

C-K, one of the most pharmacologically effective minor ginsenosides, has anti-allergic, anti-diabetic, anti-inflammatory, anti-tumor, and hepatoprotective effects [3]. Since C-K is absent in natural ginseng, it can be transformed from the glycoside hydrolysis of major PPDGs like Rb1, Rb2, Rc, and Rd. The production of C-K from PPDGs in ginseng extract has been attempted through fermentation [4-11] and biotransformation using cells [12] and recombinant [13], commercial [14-16], and wild-type enzymes [17]. The biotransformation using recombinant enzymes shows the highest yield, selectivity, and productivity for C-K production [13], however, the produced C-K is debatable on food-safety problems.

The use of enzymes originated from “generally recognized as safe (GRAS)” microorganisms is the proper way to solve food safety problems. GRAS microorganisms typically include lactic acid bacteria and some fungi that have been used for the manufacture of products in the food industry for a long time. Fungi are more suitable for C-K production than lactic acid bacteria because they are easier to grow in cheaper mediums and exhibit higher productivity [2]. Fermentation using GRAS fungi is one of the most popular methods of C-K production. However, this method results in the formation of unnecessary by-products that can cause a problem in the product recovery. Moreover, fermentation exhibits low productivity owing to prolonged cultivation time [1]. Although the productivity of C-K produced by extracellular enzymes from GRAS fungi is higher than that by fermentation, the productivity is still quite low.

In this study, we aimed to overcome the current disadvantages of C-K production such as food safety problems of recombinant enzymes and low productivities of enzymes from GRAS microorganisms. For those purposes, we chose *Aspergillus niger* KACC 46495 as a compound K-producing strain and added carboxymethyl cellulose (CMC) into the medium for inducing effective glycoside-hydrolyzing extracellular enzymes. We applied extracellular enzyme from *A. niger* to the biotransformation of PPD-type ginsenosides in Korean ginseng extract into C-K with high productivity.

## Materials and Methods

### Materials

The PPDG standards such as Rb1, Rb2, Rc, Rd, compound Mc-1 (C-Mc-1), compound O (C-O), F2, compound Mc (C-Mc), compound Y (C-Y), and C-K (≥ 98.0% purity) were provided from Ambo Laboratories (Republic of Korea). The PPDG mixture from Korean ginseng (PPDKG) was purchased from Ace EMzyme (Republic of Korea). The extract from 4-year-old Korean ginseng roots was purified using a HP-20 resin for the enrichment of PPDGs. The detailed extraction method was previously described [18], and the partial purified ginsenosides were used as PPDKG. The *p*-nitrophenols (*p*NPs) and o-nitrophenols (*o*NPs), including *o*NP-*β*-D-glucopyranoside, *o*NP-*β*-D-xylopyranoside, *p*NP-*α*-D-galactopyranoside, *p*NP-*β*-D-galactopyranoside, *p*NP-*α*-D-glucopyranoside, *p*NP-*β*-D-glucopyranoside (*p*NP-glu), *p*NP-*α*-L-arabinofuranoside, *p*NP-*α*-L-arabinopyranoside, *p*NP-*β*-D-xylopyranoside, and *p*NP-*β*-D-rhamnopyranoside were purchased from Sigma-Aldrich (USA). Potato dextrose broth was bought from Difco (Miller, Becton Dickinson; USA). The other reagents used were of analytical grade, obtained from commercial sources. Fungi were received by the Korean Agricultural Culture Collection (KACC; Republic of Korea)

### Medium, Culture Conditions, and Enzyme Preparation

The culture medium contained 20 g/l polysaccharide such as CMC, cellulose, ginseng powder, pectin from citrus, sugar beet sludge, or wheat bran, 10 g/l corn steep solid, 2 g/l KH_2_PO_4_, 0.3 g/l MnSO_4_·H_2_O, 0.3 g/l CaCl_2_, 5mg/l FeSO_4_·7H_2_O, 3.7 mg/l C°Cl_2_·6H_2_O, 1.4 mg/l ZnSO_4_·7H_2_O, and 1.3 mg/l MnSO_4_·H_2_O. The initial pH was 5.0. All fungi including *A. niger* KACC 46495 were incubated on potato dextrose agar for 7 days. After growing, the spores were harvested by adding sterile distilled water supplemented with 0.1% (v/v) Triton X-100 purchased from Sigma Aldrich. The spore suspension was filtered through two layers of sterilized gauze. The spores of fungus were counted by a hemocytometer (INCYTO, Republic of Korea). The fungal spores were added to 5 ml of potato dextrose broth to achieve a final concentration of 1.0 × 106 spores/ml. The suspension in a glass tube was cultivated with shaking at 26°C and 150 rpm for 1 day. After pre-culture in the glass tube, the mycelia were collected by centrifugation at 4°C at 13,000 ×*g* for 10 min and throw out the supernatant. The collected mycelia were washed out with 0.85% (w/v) of saline solution for removing potato dextrose broth by centrifugation at 4°C at 13,000 ×*g* for 10 min. The washed mycelia were added to 100 ml of the culture medium in 500 ml-baffled Erlenmeyer flasks, and the inoculated culture was then incubated for 6 days with shaking at 26°C and 150 rpm.

The culture broth (100 ml) was filtered, and solid ammonium sulfate was added to the filtrate with initially up to 30% and eventually 80% saturation. The precipitate was collected by centrifugation at 4°C at 13,000 ×*g* for 20 min. The collected precipitate was dissolved in 0.2 M citrate-phosphate buffer (pH 5.5), and the solution was dialyzed using a dialysis tube and concentrated using a centricon with 10 kDa cut-off size (Amicon Ultra-15, Millipore; USA). The concentrated solution of protein was used as the extracellular enzyme after its protein content was quantified using the Bradford assay.

### Effect of Inducer

The effect of the inducer added during cultivation on the C-K-producing activity of the extracellular enzyme was examined after cultivating the fungus for 6 days within 100 ml of the culture medium using a 500 ml-baffled flask. The inducer was 20 g/l of a polysaccharide such as CMC, cellulose, ginseng powder, pectin from citrus, sugar beet sludge, or wheat bran. The optimal concentration of CMC for obtaining extracellular enzyme with effective C-K-producing activity was determined by varying the concentration from 3 to 20 g/l. The reactions were carried out at 50°C for 6 h in 0.2 M citrate-phosphate buffer (pH 5.5) containing 1.0 mg/ml extracellular enzyme and 0.4 mg/ml of each ginsenoside (Rb1, Rb2, or Rc). The specific C-K-producing activity was defined as the amount of produced C-K/reaction time/amount of reacted enzyme. Thus, total C-K-producing activity referred to be multiplied by the amount of produced enzyme and the C-K-producing activity.

### Properties of the Extracellular Enzyme

Unless otherwise noted, the reaction was performed containing 0.4 mg/ml ginsenoside in 0.2 M citrate-phosphate buffer (pH 5.0) at 55°C for 10 min as the optimum conditions. The glycoside-hydrolyzing activity of extracellular enzyme from *A. niger* was measured after the reaction under the above conditions. One-unit (U) of the glycoside-hydrolyzing activity by the extracellular enzyme was its amount required to decrease 1 μmol each ginsenoside Rb1/min. A decrease of ginsenoside Rb1 was measured using high-performance liquid chromatography (HPLC) system (Agilent 1100;).

The effects of temperature and pH on the glycoside-hydrolyzing activity of ginsenoside Rb1 were investigated by varying the temperature from 40 to 65°C, at pH 5.5 and the pH value from 4.0 to 6.5 at 55°C for 10 min, respectively. The temperature stability of the extracellular enzyme was determined by measuring the residual activity after incubating in 0.2 M citrate-phosphate buffer (pH 5.0) at different temperatures from 40 to 65°C for 24 h. The pH stability was examined at 55°C for 24 h by measuring the residual activity after incubating the extracellular enzyme at different pH values from 4.0 to 6.5. The residual activity was decided after reacting the extracellular enzyme with ginsenoside Rb1 at 55°C and pH 5.0 for 10 min.

Activity of *β*-glucosidase was assayed using *p*NP-glu as a substrate. A reaction mixture (0.2 ml) in 0.2 M citrate-phosphate buffer (pH 5.5) containing 0.1 ml of *p*NP-glu (2 mM solution) and 0.1 ml of enzyme solution with 0.002 mg enzyme was incubated at 55°C for 10 min. The reaction was terminated by adding 0.02 ml of 2 M Na_2_CO_3_ solution. The activity was determined by measuring the increase of *p*NP or *o*NP using a UV-visible spectrophotometer in absorbance at 405 nm. The amount of released *p*NP was quantified using the concentration plot of the *p*NP and *o*NP standards. To identify the substrate specificity using same assay methods, crude enzyme preparation was assayed using 1 mM of *p*NP or *o*NP glycosides with *α*- and *β*-configuration, and the activity was indicated as the relative value to that of *p*NP-glu.

### Biotransformation of PPDGs into Compound K

To investigate the hydrolyzing pathways and biotransformation of ginsenoside Rb1, Rb2, and Rc into C-K by the extracellular enzyme, the reactions were conducted at 55°C and pH 5.0 with 1.0 mg/ml of each ginsenoside and 2.5 mg/ml extracellular enzyme for 2 or 24 h.

 To determine the optimal concentrations of substrate for C-K production, the concentrations of PPDGs in PPDKG were varied at 55°C and pH 5.0 for 12 h from 0.5 to 10.0 mg/ml (from 0.47 to 9.4 mM) with 1.0 mg/ml (1.8 U/ml) of the extracellular enzymes. The optimal concentration of the extracellular enzymes was determined by varying the concentration from 0.5 to 10.0 mg/ml (from 0.9 to 18.0 U/ml) with PPDKG as a substrate, respectively. The time-course reactions for the biotransformation of PPDGs to C-K were performed at 55°C for 18 h in 0.2 M citrate-phosphate buffer (pH 5.0) containing 8.0 mg/ml (14.4 U/ml) of the extracellular enzyme and 6.0 mg/ml (5.6 mM) of PPDGs in PPDKG.

### Analytical Methods

The reaction mixture was extracted by adding the equal volume of n-butanol supplemented with 1.0 mg/ml digoxin as an internal standard. The fraction containing n-butanol was dried, and the residue was dissolved in methanol. The ginsenosides were analyzed using the HPLC system with an octadecylsilica column and a UV detector at 203 nm. The column was eluted at 40°C by ginsenoside solution containing digoxin with a linear gradient of solvents such as acetonitrile/water (v/v) from 30:70 to 60:40 for 20 min, 60:40 to 90:10 for 10 min, 90:10 to 30:70 for 5 min, and at a constant 30:70 for 10 min with a flow rate of 1 ml/min. All ginsenosides, including reagent ginsenosides, biotransformed ginsenosides, and ginsenosides in PPDGs, biotransformed PPDGs, PPDKG, and biotransformed PPDKG, were quantified by the calibration curves using the ginsenoside standards.

## Results and discussion

### Effect of Inducer Added during Cultivation on the C-K-Producing Activity of the Extracellular Enzyme for Ginsenosides Rb1, Rb2, and Rc

For finding a fungus that produces an effective glycoside-hydrolyzing enzyme, extracellular enzymes from a hundred of fungi were screened by measuring their C-K-producing activities for ginsenosides Rb2 and Rc. The extracellular enzyme from *A. niger* KACC 46495 showed the highest C-K-producing activity among the fungal extracellular enzymes tested (data not shown), and it was then used for further investigation of C-K production.

The polysaccharide as a carbon source in the medium during cultivation of fungus is known to effectively induce the glycoside-hydrolyzing enzyme. For an example, wheat bran is used to induce the enzymatic activity of exoglucanase from *A. niger* [19]. To induce the C-K-producing enzyme from *A. niger* KACC 46495, different-type polysaccharide such as carboxymethyl cellulose (CMC), cellulose, ginseng powder, sugar beet sludge, pectin from citrus, or wheat bran was added to the culture medium during cultivation. When CMC was added to the medium, the specific C-Kproducing activities for ginsenoside Rb1, Rb2, or Rc were the highest amongthe inducers tested. Although the total C-K-producing activity using ginseng powder was higher than that using CMC, CMC was decided as an optimal inducer because the enzyme with higher specific activity was a more efficient biocatalyst. The optimal concentration of CMC was 10 g/l (Fig. S1).

### Biochemical Properties of the Extracellular Enzyme for Ginsenosides

The effects of temperature and pH on the glycoside-hydrolyzing activity of the extracellular enzyme were evaluated by the decrease of ginsenoside Rb1 as a substrate. Although the glycoside-hydrolyzing activity was a maximum at 60°C within 10 min, the residual activity significantly decreased after the reaction of the extracellular enzyme above 60°C for 24 h (Fig. S2A). The activity and stability were maximal at pH 5.0 (Fig. S2B). Therefore, the reaction temperature and pH for C-K production were determined as 55°C and 5.0, respectively.

Since the extracellular enzymes of fungi seemed to contain various enzymes, it was difficult to define exactly enzyme involved in the hydrolysis of specific ginsenoside without fractionation and high-purity refining of enzymes. Although enzymes were not defined exactly, the hydrolytic properties of extracellular enzyme for various sugars could be determined using *p*NP or *o*NP assay. We performed the *p*NP or *o*NP assay for extracellular enzyme from *A. niger* KACC 46495 and the activities for aryl-glycosides followed the order *p*NP-*α*-L-arabinofuranoside > *p*NP-*α*-D-galactopyranoside > *p*NP-*β*-D-glucopyranoside > *o*NP-*β*-D-xylopyranoside > *p*NP-*β*-D-xylopyranoside > *p*NP-*β*-D-galactopyranoside > *p*NP-*α*-D-glucopyranoside (Table S1). The relative activities of *o*NP-*β*-D-glucopyranoside, *p*NP-*α*-L-arabinopyranoside, and *p*NP-*β*-D-rhamnopyranoside were less than 6% to that of *p*NP-*β*-D-glucopyranoside. These results suggested that the main enzyme of the extracellular enzyme was *α*-L-arabinofuranosidase.

The glycoside-hydrolyzing activity of the extracellular enzyme for PPDGs as substrates followed the order Rb1 C-Mc1 > C-O > Rc > Rd > Rb2 > C-Mc > F2 > C-Y, but no activity was found for C-K (Table 1). The glycoside-hydrolyzing activity of the extracellular enzyme for major PPDGs followed the order Rb1 > Rc > Rd > Rb2. This order was the same as those for purified enzymes from *Penicillium oxalicum*, *P. aculeatum*, and *Paecilomyces bainier* [20-22]. The extracellular enzymes from fungi seem to exhibit the lowest hydrolyzing activity for Rb2 among other major PPDGs.

### Biotransformation of Ginsenoside Rb1, Rb2, and Rc into Compound K by the Extracellular Enzyme

**Fig. 2:**
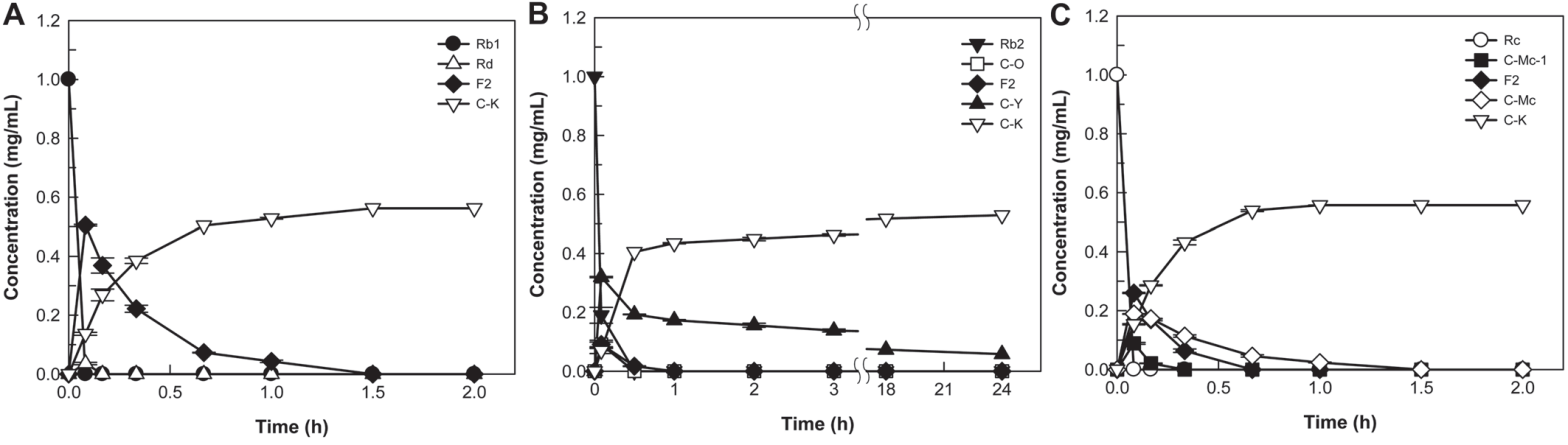
Biotransformation of ginsenosides (A) Rb1, (B) Rb2, and (C) Rc into compound K by extracellular enzyme from *A. niger*. The extracellular enzyme from *A. niger* converted 1.0 mg/ml of ginsenoside Rb1, Rb2, and Rc into C-K with molar conversions of 100%, 94%, and 100%, respectively. Data are expressed as the means of three experiments and the error bars represent standard deviations.

The reactions of time-course for the biotransformation of ginsenoside Rb1, Rb2, and Rc into C-K were conducted at 55°C and pH 5.0. The extracellular enzyme from *A. niger* completely converted 1.0 mg/ml of ginsenoside Rb1 and Rc into C-K within 2 h, whereas the enzyme converted 1.0 mg/ml ginsenoside Rb2 into 0.53 mg/ml C-K after 24 h, with a molar conversion of 94% (Fig. 2). In the conversion of ginsenoside Rb2, the conversion rate of C-Y to C-K was very slow, which acted as a limiting step. The HPLC profiles during the conversion of ginsenosides Rb1, Rb2, and Rc into C-K by extracellular enzyme from *A. niger* are shown in Fig. S3.

The extracellular enzymes from *Armillaria mellea* [23, 24], *A. niger* KCTC 6906 [25, 26], and *A. usamii* [25, 26] did not completely convert Rb1 and Rc into C-K; they converted Rb2 into C-K with a molar conversion of less than 40% [26, 27], which was lower than those for Rb1 and Rc. These results indicate that the extracellular enzyme from *A. niger* KACC 46495 is a more efficient biocatalyst to produce food-available C-K than other reported extracellular enzymes from fungi.

### Glycoside-Hydrolyzing Pathways of PPD-Type Ginsenosides into Compound K by the Extracellular Enzyme

**Fig. 3:**
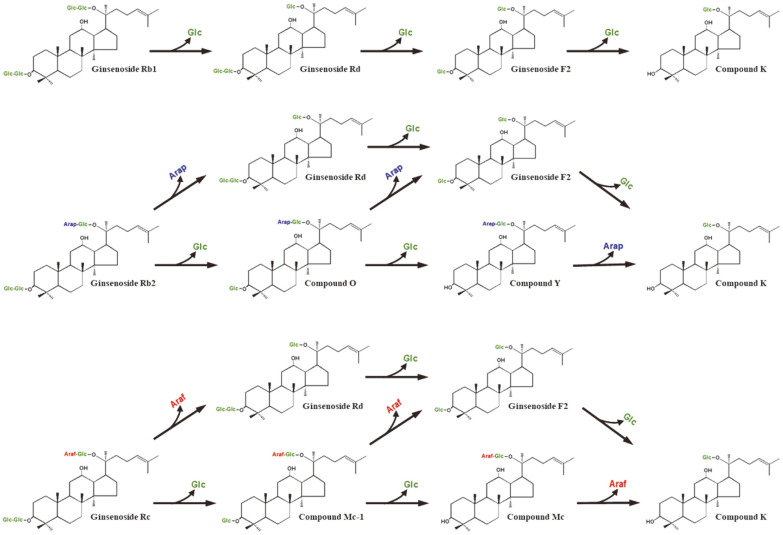
Proposed biotransformation pathways of protopanaxadiol-type ginsenosides into compound K by extracellular enzyme from *Aspergillus niger*. Glc, glucose, Arap, arabinopyranose, and Araf, arabinofuranose.

Ginsenosides Rb1, Rb2, and Rc typically have glucose molecules at C3 and C20, however, the outer monosaccharide portions at C20 in the ginsenosides contain different monosaccharides such as glucose, arabinopyranose, and arabinofuranose, respectively. The intermediates of the conversion of Rb1, Rb2, and Rc as substrates into C-K by extracellular enzyme from *A. niger* were identified through the time-course reactions (Fig. 2) and HPLC profiles (Fig. S3). Ginsenoside Rb1 was enzymatically converted into C-K via Rd and F2 (Fig. S3A). Ginsenoside Rb2 or Rc was converted not only into Rd by the hydrolysis of the outer arabinopyranose or arabinofuranose of C-20 but also into C-O or C-Mc1 by the hydrolysis of the outer glucose of C-3, respectively. The formed C-O or C-Mc1 was converted into F2 and C-Y or C-Mc by the hydrolysis of the outer arabinopyranose or arabinofuranose at C-20 and the inner glucose at C-3, respectively (Fig. S4). Eventually, these ginsenosides were converted into C-K by the hydrolysis at C-3 and C-20 excluding the inner glucose at C-20. Thus, the biotransformation pathways of PPDGs into C-K by extracellular enzyme from *A. niger* KACC 46495 were Rb1 → Rd → F2 → C-K, Rb2 → Rd or C-O → F2 or C-Y → C-K, and Rc → Rd or C-Mc1 → F2 or C-Mc → C-K (Fig. 3).

The biotransformation pathways of PPDGs into C-K by extracellular enzymes from *A. niger* g.848 and *A. niger* Z229 were the same as those by *A. niger* KACC 46495 [6, 28]. However, the pathways by extracellular enzyme from *A. niger* g.48 for Rb1 showed not only Rb1 → Rd → F2 → C-K but also Rb1 → Gypenoside XVII → Gypenoside LXXV C-K [29]. The extracellular enzyme from *A. niger* g48p exhibited the pathway of Rb1, Rb2, or Rc → F2→ C-K or Rh2 [30].

### Biotransformation of PPD-type ginsenoside mixture from Korean ginseng extract into compound K by the extracellular enzyme

PPDKG contained 47.3% (w/w) PPDGs, including Rb1 (165 mg/g), Rc (100 mg/g), Rb2 (117 mg/g), and Rd (91 mg/g). The optimal concentration of PPDGs in PPDKG for C-K production was decided by varying the concentration from 0.5 to 10.0 mg/ml with 1.0 mg/ml extracellular enzyme. C-K production was reached 100%conversion yields when treating 0.5 mg/ml of PPDGs and the amount of produced compound K was steadily increased with a rise in the concentration of PPDGs. However, the rate of C-K production above 6.0 mg/ml decreased slightly (Fig. S5A). To determine the optimal concentration of the extracellular enzyme, the concentration was varied from 0.5 to 10.0 mg/ml with 6.0 mg/ml PPDGs in PPDKG. C-K production increased as the concentration of extracellular enzyme increased, however, C-K production above 8.0 mg/ml reached a plateau (Fig. S5B). Thus, the optimal concentrations of PPDGs and extracellular enzyme for C-K production were 6.0 mg/ml and 8.0 mg/ml, respectively.

**Fig. 4:**
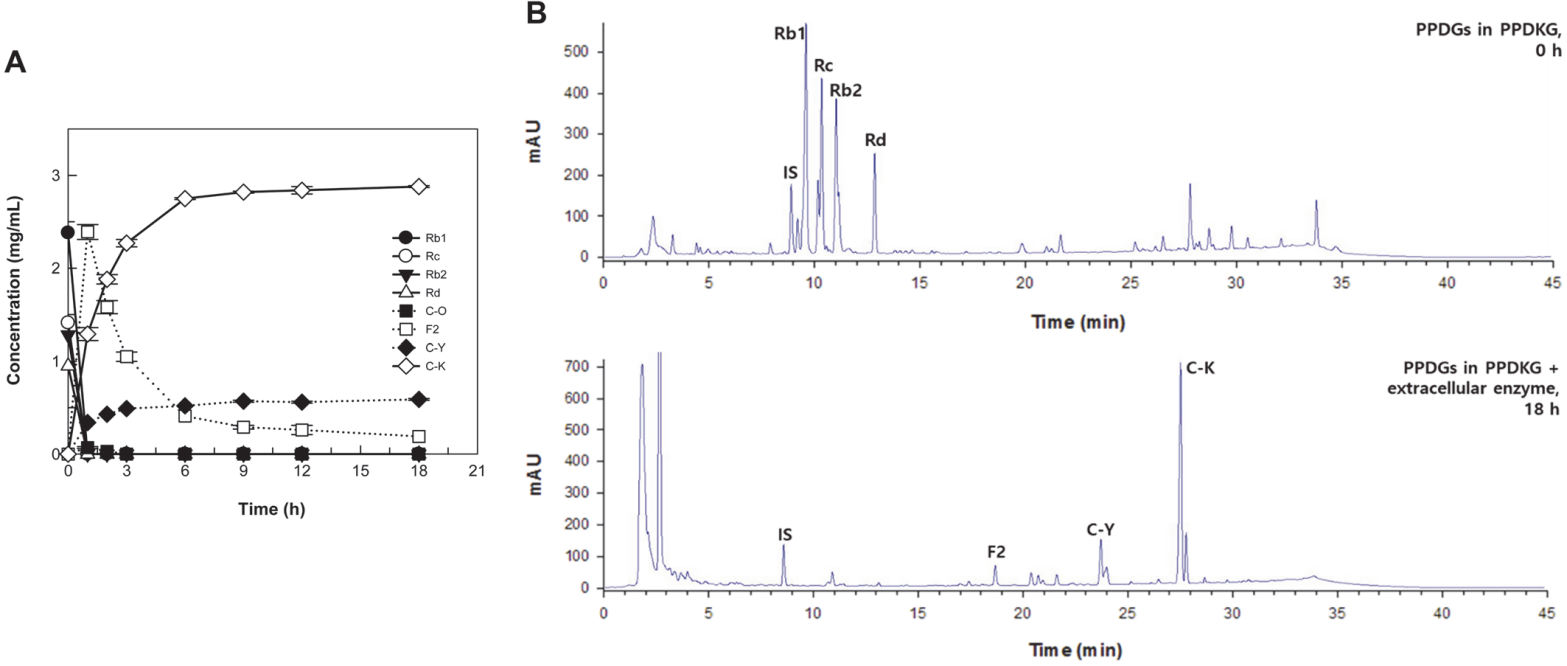
Biotransformation of protopanaxadiol-type ginsenosides (PPDGs) in protopanaxadiol-type ginsenoside mixture from Korean ginseng (PPDKG) into compound K by extracellular enzyme from *A. niger*. (A) Time-course reactions of protopanaxadiol-type ginsenosides into compound K. (B) HPLC profiles before and after the reaction. ●, Ginsenoside Rb1; ○, ginsenoside Rc; ▼ , ginsenoside Rb2; △, ginsenoside Rd; ■ , compound O; □ , ginsenoside F2; ◆, compound Y; ◇, compound K. Data are expressed as the means of three experiments and the error bars represent standard deviations.

The time-course reactions for the biotransformation of PPDGs in PPDKG into C-K were performed for 18 h under the optimized conditions of 55°C, pH 5.0, 6.0 mg/ml PPDGs, and 8.0 mg/ml extracellular enzyme. The extracellular enzyme converted 6.0 mg/ml (5.6 mM) PPDGs in PPDKG into 2.8 mg/ml (4.5 mM) C-K in 9 h, with a productivity of 313 mg/l/h and a molar conversion of 80% (Fig. 4). The biotransformation of PPDGs into C-K by crude enzymes from food-available wild-type microorganisms is presented in Table 2. The commercial enzyme Cytolase PCL5 converted white ginseng extract into 2.1 mg/ml C-K in 78 h, with a productivity 26.9 mg/l/h, which was the previously highest recorded concentration and productivity [14]. The concentration and productivity of C-K by extracellular enzyme from *A. niger* KACC 46495 were 1.3- and 11.6-fold higher than that by Cytolase PCL5, respectively.

In conclusion, the biochemical properties of extracellular enzyme from *A. niger* were characterized, and the induction and reaction conditions for C-K production were optimized. Under the optimized conditions, the induced extracellular enzyme from *A. niger* effectively converted PPDGs of Korean ginseng extract into food-available C-K. To the best of our knowledge, this is the highest productivity and concentration of C-K among those produced by crude enzymes from wild-type microorganisms thus far. Therefore, our results will be helpful to the industrial production of food-available C-K by biotransformation.

## Supplemental Materials



Supplementary data for this paper are available on-line only at http://jmb.or.kr.

## Figures and Tables

**Fig. 1:**
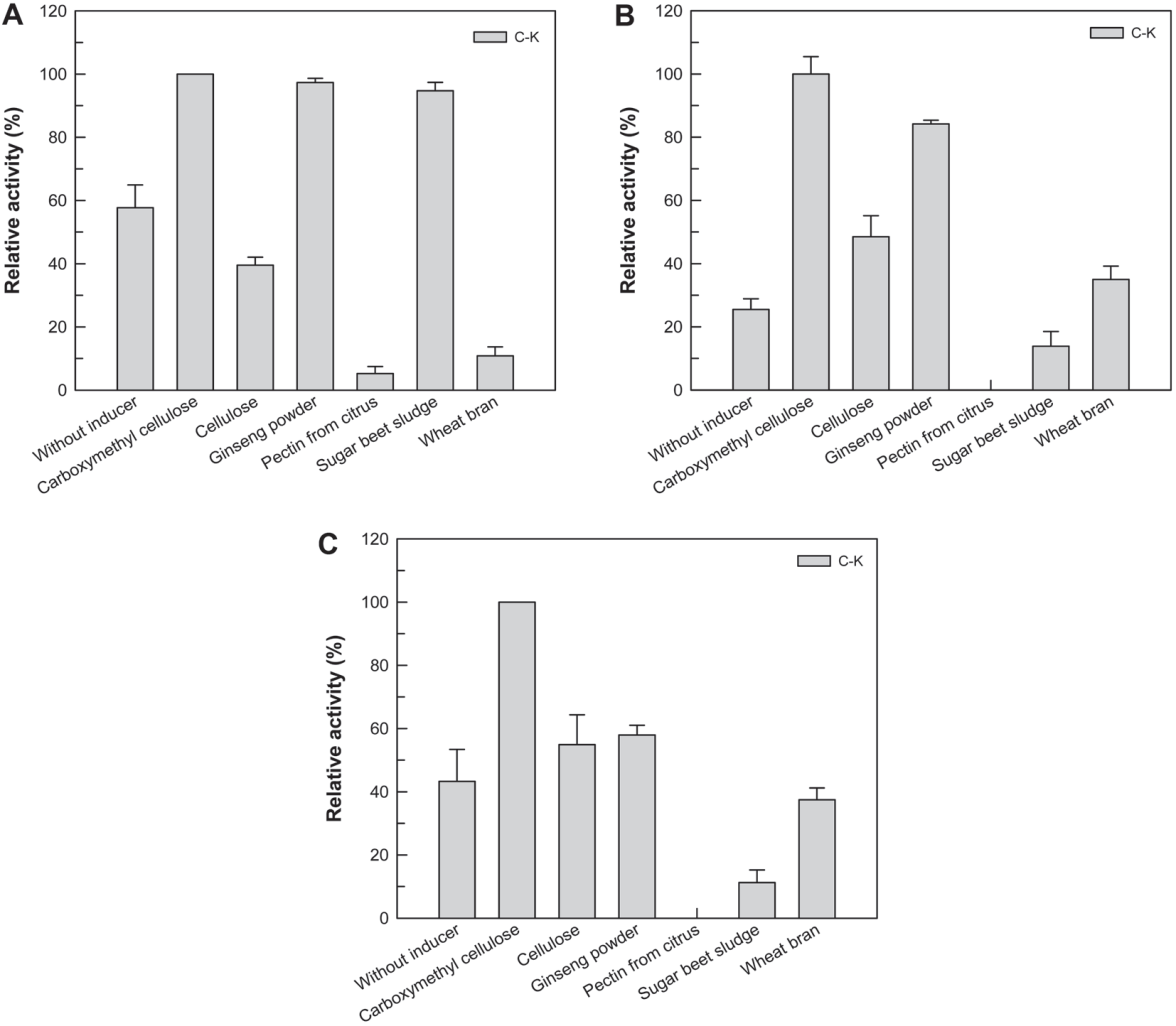
Effect of inducer added during cultivation on the hydrolytic activity for ginsenosides (A) Rb1, (B) Rb2, and (C) Rc. The inducer polysaccharide at 20 g/l was added during cultivation. Data are expressed as the means of three experiments and the error bars represent standard deviations.

**Table 1:** Hydrolytic activity of extracellular enzyme from *A. niger* for PPDGs.

Ginsenoside	Hydrolytic activity (nmol/min/mg)
Rb1	1769.0 ± 45.50
Rb2	97.7 ± 4.41
Rc	225.5 ± 7.62
Rd	156.5 ± 1.25
C-Mc1	484.9 ± 1.10
C-O	376.5 ± 1.10
F2	73.1 ± 0.87
C-Mc	78.6 ± 0.08
C-Y	11.6 ± 0.16
C-K	0.00 ± 0.00

Data are expressed as the means of three experiments and the error bars represent standard deviations.

**Table 2:** Biotransformation of PPDGs to C-K by crude enzymes from food-available wild-type microorganisms.

Origin of enzyme	Microorganism	Substrate	C-K (mg/mL)	Molar conversion (%)	Productivity (mg/L/h)	Reference
Commercial enzyme	*Aspergillus niger* (Cytolase PCL5)	White ginseng extract	2.1	69	26.9	[14]
	*Aspergillus niger* (Pectinex)	Non-extracted raw rootlet ginseng	1.0	NC	13.7	[16]
Lactic acid bacteria	*Bifidobacterium* sp. Int57	Rb1	0.5	97	11.3	[25]
		Rb2	0.2	94	11.0	
		Rc	0.4	77	9.2	
	*Bifidobacterium* sp. SJ32	Rb1	0.5	94	11.0	
		Rb2	0.5	87	10.2	
		Rc	0.4	48	7.3	
	*Bifidobacterium minimum* KK-1	Rb1	0.003	5.4	0.1	[17]
		Rb2	0.004	6.4	0.2	
		Rc	0.001	2.3	0.04	
		Ginseng extract	0.007	NC	0.3	
	*Bifidobacterium cholerium* KK-2	Rb1	0.0004	0.8	0.02	
		Rb2	0.0007	1.2	0.03	
		Rc	0.0006	1.1	0.03	
		Ginseng extract	0.004	NC	0.2	
	*Leuconostoc lactis* DC201	Rb1	0.6	99	7.8	[31]
	*Leuconostoc mesenterioides* DC102	Rb1	0.6	99	7.8	[32]
	*Lactobacillus paralimentarius* LH4	Rb1	0.5	88	7.2	[33]
Fungi	*Armillaria mellea* KACC 50013	Rb1	0.1	100	5.8	[23]
	*Aspergillus niger* KCTC 6906	Rb1	0.5	96	11.3	[25]
		Rb2	0.05	9.7	1.0	[26]
		Rc	0.05	9.1	1.0	
	*Aspergillus usamii* var. *shirousamii* KCTC 6956	Rb1	0.3	55.2	6.5	[25]
		Rb2	0.06	10.4	1.3	[26]
		Rc	0.3	52	6.1	
	*Aspergillus niger* KACC 46495	Rb1	0.6	100	280	This study
		Rb2	0.5	94	22	
		Rc	0.6	100	289	
		PPDKG	2.8	80	313	

NC, Not calculated.
