# Supplementary material for: Biotransformation of Protopanaxadiol-Type Ginsenosides in Korean Ginseng Extract into Food-Available Compound K by an Extracellular Enzyme from Aspergillus niger
Source: J Microbiol Biotechnol. 2020 Jul 31;30(10):1560–7. doi: 10.4014/jmb.2007.07003 (PMC9728230; doi:10.4014/jmb.2007.07003)
Supplement: Supplementary file 1 [file JMB-30-10-1560-supple.pdf]

J. Microbiol. Biotechnol.
https://doi.org/10.4014/jmb.2007.07003

Supporting information

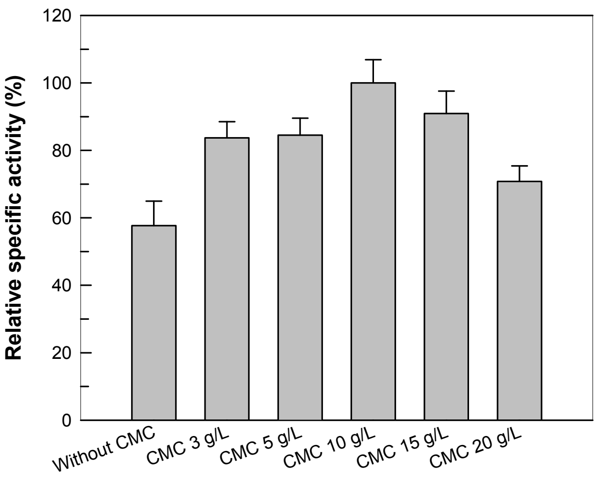

Fig. S1. Effect of the concentration of carboxymethyl cellulose added during cultivation on the hydrolytic activity for ginsenoside Rb2. The reactions were performed at 50°C for 6 h in 0.2 M citrate-phosphate buffer (pH 5.5) containing 0.4 mg/mL ginsenoside Rb2 and 1.0 mg/mL extracellular enzyme. Data are expressed as the means of three experiments and the error bars represent standard deviations.

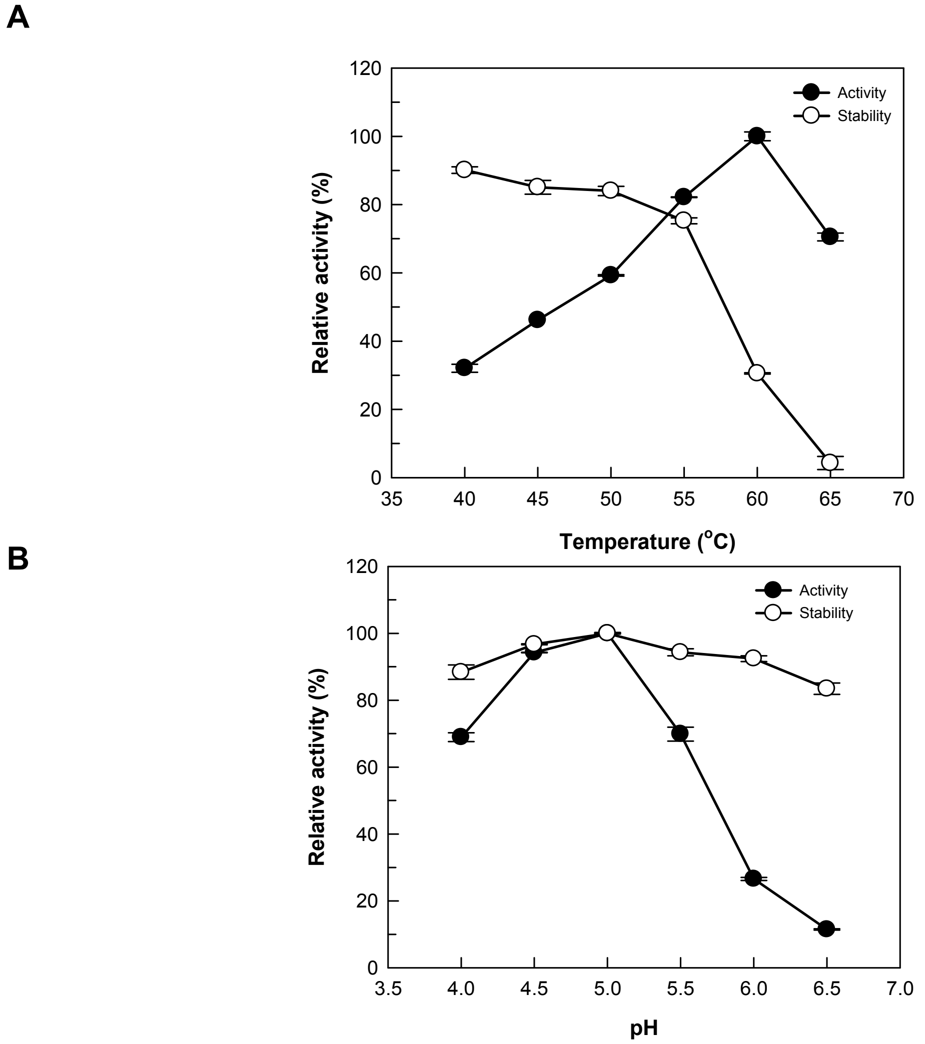

Fig. S2. Effects of temperature and pH on the activity and stability of extracellular enzyme from *Aspergillus niger* for ginsenoside Rb1. (A) Temperature effect. The reactions were performed for 10 min in 0.2 M citrate-phosphate buffer (pH 5.0) containing 0.4 mg/mL ginsenoside Rb1 and 1.0 mg/mL extracellular enzyme by varying the temperature from 40 to 65°C. (B) pH effect. The reactions were performed at 55°C for 10 min in 0.2 M citrate-phosphate buffer containing 0.4 mg/mL ginsenoside Rb1 and 1.0 mg/mL extracellular enzyme by varying the pH 4.0 to 6.5. The reactions of temperature and pH stability of the extracellular enzyme were determined by measuring the residual activity at 55°C and pH 5.0 after incubating the extracellular enzyme for 24 h. Data are expressed as the means of three experiments and the error bars represent standard deviations.

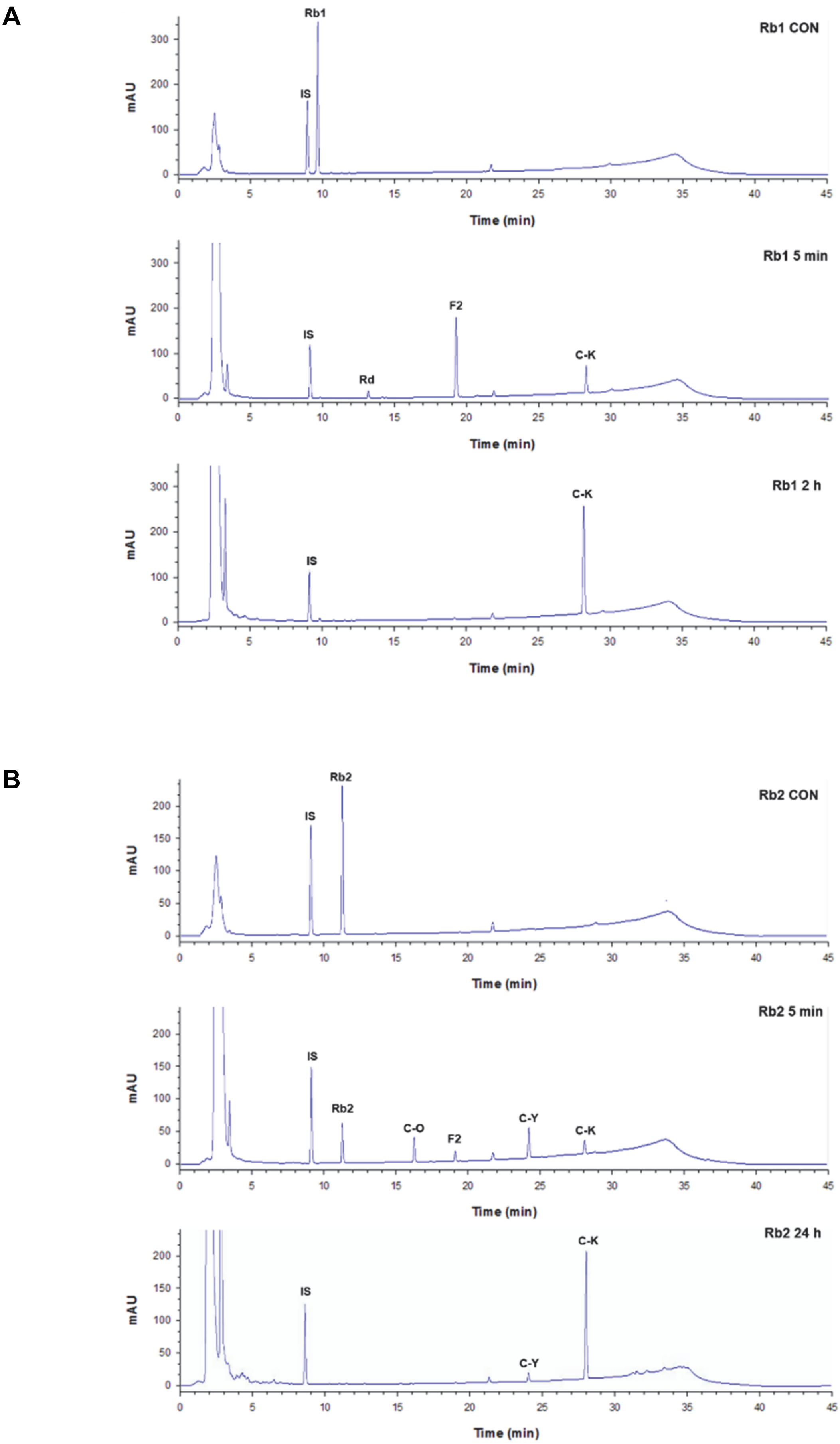
A
Rb1 CON
Rb1
IS
mAU
Time (min)
Rb1 5 min
IS
Rd
F2
C-K
Rb1 2 h
C-K
IS
B
Rb2 CON
Rb2
IS
Rb2 5 min
IS
Rb2
C-O
F2
C-Y
C-K
Rb2 24 h
C-K
IS
C-Y

C

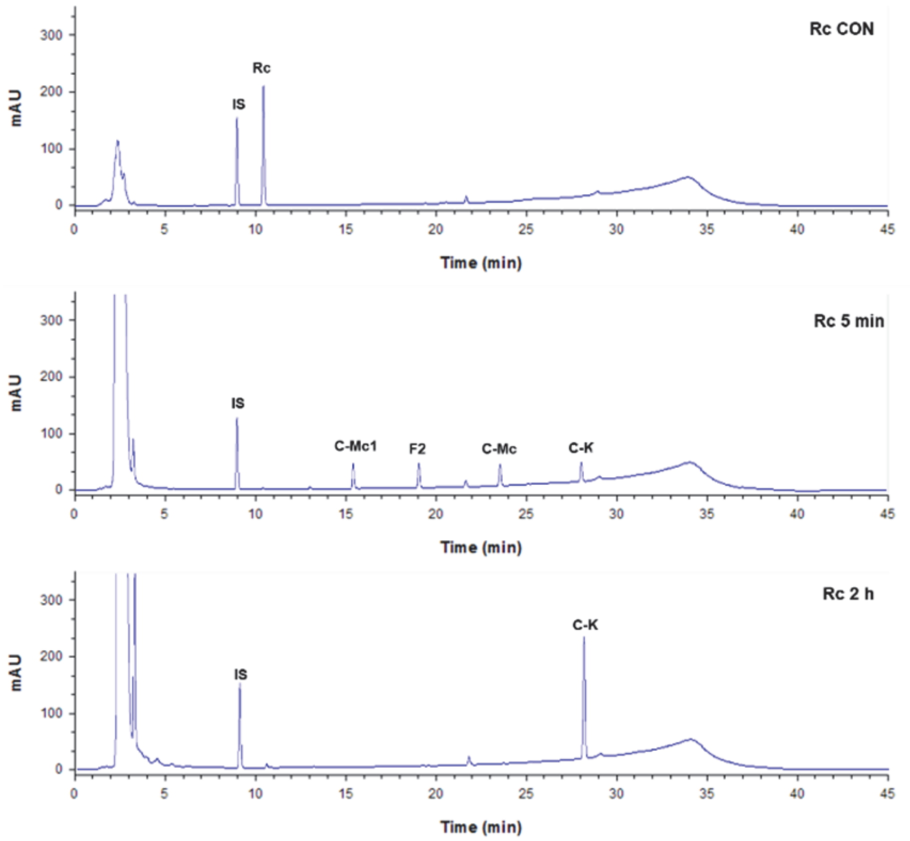

Fig. S3. High-performance liquid chromatography profiles during the conversion of ginsenosides Rb1, Rb2, and Rc into compound K by extracellular enzyme from *Aspergillus niger*. (A) Conversion of Rb1 to compound K (C-K). (B) Conversion of Rb2 to C-K. (C) Conversion of Rc to C-K. The reactions were performed at 55°C for 5 min and 2 h or 24 h in 0.2 M citrate-phosphate buffer (pH 5.0) containing 1.0 mg/mL of each ginsenoside and 2.5 mg/mL extracellular enzyme. Control (CON) indicated high-performance liquid chromatography profile of substrate at initial time.

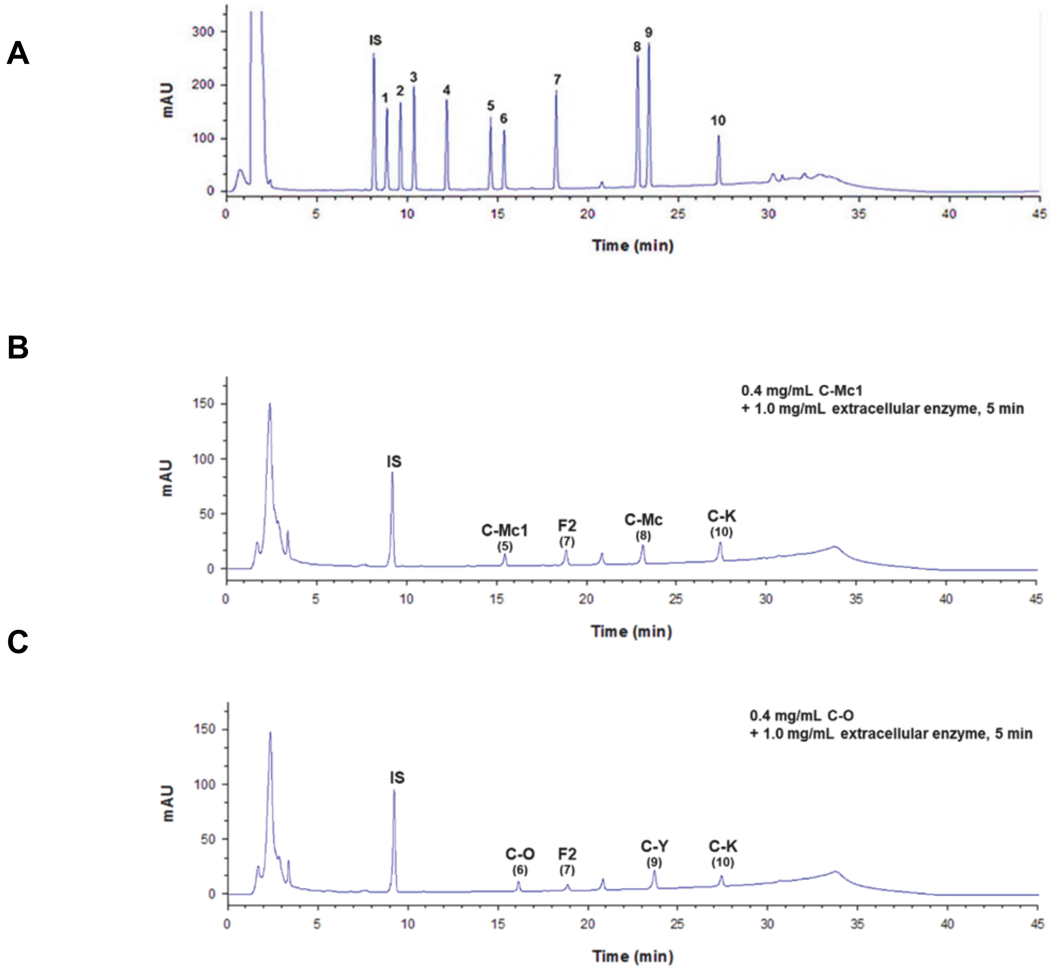

Fig. S4. High-performance liquid chromatography profiles for the conversion of compound O and compound Mc1 into compound K by extracellular enzyme from *Aspergillus niger* for 5 min with PPD-type ginsenoside standards. (A) High-performance liquid chromatography (HPLC) profile for ginsenoside standards. (B) HPLC profile for reaction products of compound Mc1 (C-Mc1). (C) HPLC profile for reaction products of compound O (C-O). The reactions were performed at 55°C for 5 min in 0.2 M citrate-phosphate buffer (pH 5.0) containing 0.4 mg/mL of C-Mc1 or C-O and 1.0 mg/mL extracellular enzyme. Internal standard (IS, digoxin); 1, Rb1; 2, Rc; 3, Rb2; 4, Rd; 5, C-Mc1; 6, C-O; 7, F2; 8, compound Mc (C-Mc); 9, compound Y (C-Y); and 10, compound K (C-K).

A

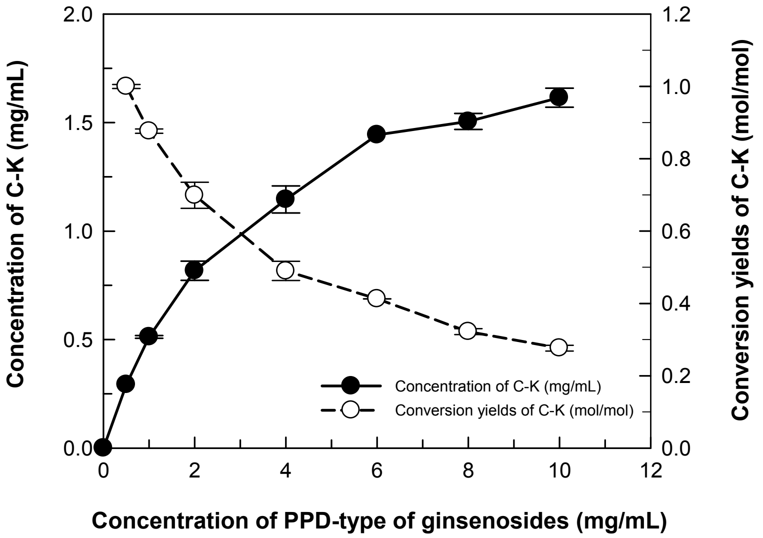

B

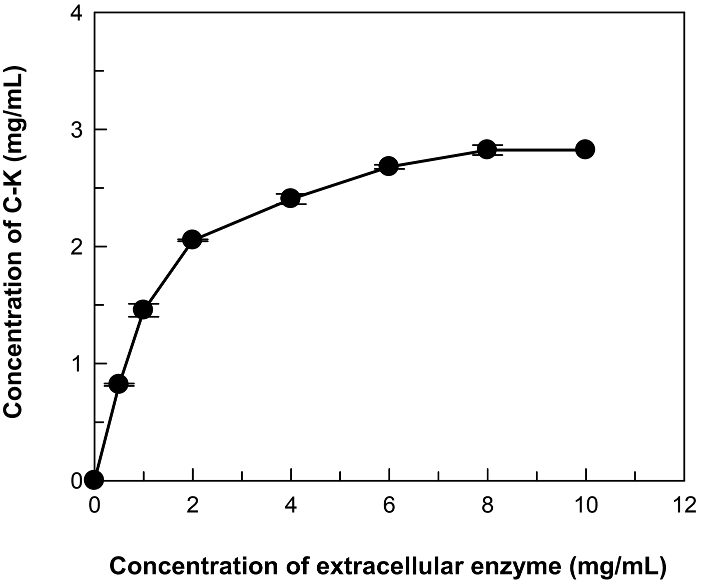

Fig. S5. Effects of the concentrations of (A) protopanaxadiol-type ginsenoside mixture from Korean ginseng (PPDKG) and (B) extracellular enzyme from *Aspergillus niger* on compound K production. (A) The reactions were performed at 55°C for 12 h in 0.2 M citrate-phosphate buffer (pH 5.0) containing 1.0 mg/mL extracellular enzyme by varying the concentration of PPDKG from 0.5 mg/mL to 10.0 mg/mL. (B) The reactions were performed at 55°C for 12 h

in 0.2 M citrate-phosphate buffer (pH 5.0) containing 6.0 mg/mL PPDKG by varying the concentration of extracellular enzyme from 0.5 mg/mL to 10.0 mg/mL. ●, Concentration of produced compound K (mg/mL); ○, Conversion yields of produced compound K (mol/mol). Data are expressed as the means of three experiments and the error bars represent standard deviations.

Table S1. Relative activity of extracellular enzymes from *A. niger* for aryl-glycosides.

Substrate[1]	Specific activity (μmol/min/mg)	Relative activity (%)
*o*NP[2]-*β*-D-glucopyranoside	0.5 ± 0.00	5.6 ± 0.03
*o*NP-*β*-D-xylopyranoside	4.2 ± 0.01	51.6 ± 0.14
*p*NP[3]-*α*-D-galactopyranoside	14.8 ± 0.33	181 ± 4.05
*p*NP-*β*-D-galactopyranoside	3.8 ± 0.04	46.4 ± 0.43
*p*NP-*α*-D-glucopyranoside	1.0 ± 0.00	12.0 ± 0.00
*p*NP-*β*-D-glucopyranoside	8.2 ± 0.07	100 ± 0.87
*p*NP-*α*-L-arabinofuranoside	18.9 ± 0.12	230 ± 1.45
*p*NP-*α*-L-arabinopyranoside	0.11 ± 0.00	1.3 ± 0.00
*p*NP-*β*-D-xylopyranoside	4.1 ± 0.01	50.4 ± 0.14
*p*NP-*β*-D-rhamnopyranoside	0.10 ± 0.01	1.3 ± 0.06

[1]Substrate concentration: 1 mM.

[2]*o*NP: *o*-nitrophenol

[3]*p*NP: *p*-nitrophenol
